# Multiple discrimination at work and depressive symptoms in young adults

**DOI:** 10.1186/s40359-026-05765-y

**Published:** 2026-10-11

**Authors:** Tobias Weinmann, Britta Herbig, Felix Forster, Laura Wengenroth, Dennis Nowak, Christian Vogelberg, Katja Radon

**Affiliations:** 1 Institute and Clinic for Occupational, Social and Environmental Medicine, LMU Medizin, LMU University Hospital, Ludwig-Maximilians-Universität München, Munich, Germany; 2 Department of Pediatrics, Faculty of Medicine, University Hospital Carl Gustav Carus, Technische Universität Dresden, Dresden, Germany

**Keywords:** Discrimination, Workplace, Mental health, Depressive symptoms, Health inequities, Occupational safety and health

## Abstract

**Background:**

We aimed to estimate the implications of cumulative exposure to discrimination at work for mental health among young adults. Our specific objective was to investigate if experiencing multiple types of discrimination at work is associated with depressive symptoms.

**Methods:**

We analyzed cross-sectional questionnaire data from 1066 participants (56.8% female, mean age 31.3 years) of the third phase of the German Study on Occupational Allergy Risks (2017–2018, response 47.3%). Participants were asked whether during the last twelve months they had experienced one or more of seven types of discrimination at their workplace. The Patient Health Questionnaire-2 (PHQ-2) was used for screening of symptoms of depression. We performed multivariate logistic regression to estimate the association between the number of experienced types of discrimination at work and depressive symptoms.

**Results:**

Discrimination due to sex was the most often reported type (6.1% of participants). Experience of two or more forms of discrimination was indicated by 2.5% of the participants. One hundred four individuals (9.8%) were screened positive for depression. Before adjustment for work stress, individuals reporting two or more types of discrimination at work had more than six times the odds of depressive symptoms compared to the non-exposed reference group (Odds Ratio (OR): 6.31, 95% Confidence Interval (CI): 2.67 to 14.89). After adjustment for work stress, the OR for the association between two or more types of discrimination and depressive symptoms was 3.92 (95% CI: 1.40 to 11.01).

**Conclusion:**

Our results support the hypothesis of an association between exposure to multiple discrimination at work and poor mental health, even though our effect estimates suffer from low statistical precision. Furthermore, the design of our analysis does not permit causal statements. In view of the entire scientific body of studies in the field, we argue that measures to reduce or abolish discrimination may be seen as important means of protecting mental health and well-being.

**Supplementary Information:**

The online version contains supplementary material available at https://doi.org/10.1186/s40359-026-05765-y.

## Background

Discrimination, defined as the unequal treatment of individuals because of their membership in stigmatized or marginalized groups, e.g., based on race or gender [1, 2], is a major social determinant of health [3, 4]. Studies have found consistent associations between exposure to discrimination and a wide range of mental disorders (e.g., depression or anxiety) as well as physical diseases such as hypertension or obesity [5–7]. Furthermore, there are reports linking discrimination with loneliness [8] as well as consumption of alcohol, tobacco and marijuana [9–13] while there is also indication that discrimination considerably contributes to health inequities [14, 15].

People’s workplace is one of the settings where they can be subjected to various forms of discrimination, e.g., through hiring or promotion practices that disadvantage minoritized groups or through being treated differently in interpersonal relationships at work because of their individual characteristics or social group membership [16–18]. In Europe for example, 5.23 million women and 3.63 million men reported experiences of discrimination at work in 2021 according to the EU labor force survey [19]. The European Working Conditions Survey (EWCS) estimated the prevalence of employees exposed to workplace discrimination across the EU at 12% [20]. In the US, a study representing more than 40 million workers estimated the prevalence of discrimination at work between 25% for black women and 11% for white men [21]. Being treated in a discriminatory fashion at work is considered to contribute to employment precariousness, a phenomenon that is currently on the rise in many countries [22, 23]. In addition, studies among the scientific and healthcare workforce indicated that discrimination can have various negative consequences for the victims including stress, job dissatisfaction, isolation or burnout [24].

Despite such reports, and even though work-related factors are growingly recognized as key social determinants of health [25], the body of studies looking at the mental health consequences of discrimination at the workplace is still rather small [26]. The few studies that exist mostly either performed a general assessment of discrimination without identifying the specific ‘type’ of discrimination in their analysis [16, 26] or concentrated on one specific type such as discrimination based on ethnicity [18, 27], age [28] or gender [11, 29]. A study that used data from the above-mentioned EWCS to explore mental well-being in relation to different aspects of psychosocial working conditions including exposure to discrimination [30] did not account for the number of reported types of discrimination in their analyses.

We hypothesize that experiencing discrimination on the grounds of multiple types (e.g., age, gender, and sexual orientation) represents a form of cumulative exposure that warrants closer study [31]. Framed within the allostatic load model outline in more detail below, an accumulation of stressors in the form of multiple discrimination may inflict repeated “hits” on the individual, accelerating the shift from allostatic load to overload and triggering systemic dysregulation [32]. Possibly, cumulative or additive exposure to various types of discrimination might also serve as a means of investigating a potential dose-response relationship between workplace discrimination and mental impairment. One study that assessed multiple types of workplace discrimination was performed among hospital employees in the US reporting evidence for an association between types and occurrence of workplace discrimination and depressive symptoms [33]. Another American study conducted semi-structured qualitative interviews with black female physicians to investigate their experience of discrimination at the intersection of ethnicity and gender [34]. Apart from such rather isolated examples, there seems to be very little research on multidimensional workplace discrimination in relation to mental health. Furthermore, the mental health implications of discrimination so far have been predominantly investigated among older adults, somewhat neglecting younger adults even though there is reason to assume that detrimental health effects of discrimination may commence early in the life course [35].

Concerning the potentially underlying mechanisms, there is indication that workplace discrimination may affect health outcomes through negative emotions, perceived injustice and/or physiological stress reactions [10, 17, 18, 36]. With respect to the latter, constant activation of the hypothalamic-pituitary-adrenocortical axis [37] as a physical reaction to an external stimulus, in this case discrimination, seems to play an important role [7]. The cost of this chronic neural and neuroendocrine response to stressful events has been defined as allostatic load [32], which can be interpreted as a dysregulation in response to stress, potentially leading to a variety of different health outcomes [38]. In fact, there is a range of studies reporting a link between experience of discrimination and allostatic load [7, 39] including observations of an association between intersectional discrimination and hair cortisol concentration as a specific marker of allostatic load [40]. Studies yielding evidence for an impact of other work-related stressors on markers of allostatic load further corroborate these considerations [41, 42].

Therefore, we aimed to expand the existing body of scientific evidence concerning the implications of experiencing discrimination at work for individuals’ mental health. Our specific objective was to investigate if experiencing multiple types of discrimination at work is associated with depressive symptoms via the following hypothesis: An increase in the number of types of experienced discrimination at work is associated with an elevated likelihood of depressive symptoms.

## Methods

### Study design and participants

We analyzed cross-sectional data from the third phase of the Study on Occupational Allergy Risks (SOLAR 3; *N* = 1359, response 47.3%) conducted in the cities of Dresden and Munich, Germany. The SOLAR study is a follow-up of the German participants of the International Study on Asthma and Allergies in Childhood (ISAAC) Phase II (1995–1996, *N* = 6399, participants aged 9–11 years) [43] who had agreed to be re-contacted for purposes of follow-up. Recruitment for ISAAC Phase II was done by community-based random sampling (Weiland et al., 1999). The first follow-up (SOLAR 1) was performed in 2002–2003 (*N* = 3785, participants aged 16–18 years), followed by SOLAR 2 in 2007–2009 (*N* = 2051, participants aged 19–24 years) [44]. SOLAR 3 was the latest follow-up carried out in 2017–2018. Participation in SOLAR 3 included answering a study questionnaire that could be completed as paper or online version (provided via LimeSurvey) and collected information on socio-demographic variables, job history, various occupational and environmental exposures, respiratory and allergic diseases as well as mental health [45]. Those SOLAR 3 participants who reported being in some form of employment (including internships) in the last twelve months prior to the survey (*N* = 1066) were included in the present analysis. As information on discrimination at work and mental health were not collected before SOLAR 3, we could not perform longitudinal analyses.

### Exposure assessment

Based on the questionnaire used in the 2010 and 2015 waves of the above-mentioned EWCS [46, 47], participants were asked the following question: ‘Over the past 12 months at work, have you been subjected to any of the following?

- Discrimination because of age.
- Discrimination because of origin, ethnic background or color.
- Discrimination because of nationality.
- Discrimination because of sex.
- Discrimination because of religion.
- Discrimination because of disability.
- Discrimination because of sexual orientation.

In the second item, we the replaced term “race” used in the original version of the ECWS with “origin”, since in German the use of the term ‘race’ appears rather inopportune.

As the primary aim of our study was to investigate cumulative exposure to discrimination, for each participant we calculated a score according to the reported number of types of discrimination. For instance, an individual indicating exposure to discrimination on the grounds of age, religion, and disability was classified into “3 types of discrimination”. Individuals reporting no experience of any of the seven forms of discrimination mentioned in the questionnaire were categorized as non-exposed (i.e., “0 types of discrimination”). Due to the limited number of individuals reporting exposure to multiple types of discrimination, for further analysis, we classified participants into three categories according to the number of items to which they gave a positive answer (0, 1, 2 or more).

### Outcome assessment

For the assessment of mental health, the 2-item version of the Patient Health Questionnaire (PHQ-2) was used [48]. The PHQ-2 consists of the first two questions of the longer PHQ-9 and is a screening tool for core symptoms of major depression by asking participants how often they have been bothered by any of the following problems over the last two weeks: (1) little interest or pleasure in doing things; (2) feeling down, depressed, or hopeless. Answers were provided on a four-point scale from 0 (not at all) to 3 (nearly every day) so that participants could reach a total score between 0 and 6 points. Based on the recommended cut-off point of ≥ 3 points (sensitivity 82.9%; specificity 90.0%, positive predictive value 38.4%) [48, 49], we dichotomized participants into two groups (depressive symptoms no vs. yes).

### Additional variables

In addition, we collected information on the following variables: Sex (female, male), educational attainment (no qualification, vocational training (vocational school), vocational training (technical college), university of applied sciences degree, university degree (Bachelor’s), university degree (Master’s), other degree, student) and current form of occupation (apprenticeship, student, employee, civil servant, self-employed, job seeker, not working for health reasons, maternity protection / parental leave). Furthermore, we measured chronic stress at work using three scales of the validated Trier Inventory for the Assessment of Chronic Stress (TICS) [50, 51]. These were the scales *work overload* (8 items, e.g., ‘too little time to execute my daily tasks’), *work discontent* (5 items, e.g., ‘the wish to change my current job’) and *pressure to perform* (9 items, e.g., ‘I have tasks to do where I am under critical observation’). For each item, participants had to indicate how often they had experienced the respective situation during the last year on a five-point scale (never, rarely, sometimes, often, very often). For the present analysis, we calculated participants’ sum score across all three scales, i.e., 22 items. Besides, information on country of birth (Germany, other) was taken from data initially provided during ISAAC Phase 2 [43].

### Statistical analysis

We performed logistic regression that included the number of types of experienced discrimination (0, 1, 2 or more types) as predictor and depressive symptoms (yes vs. no) as outcome. Based on the scientific literature, we a priori decided to add sex (female vs. male), study center (Dresden vs. Munich), educational achievement (university degree vs. no university degree), current form of occupation (formally employed (i.e., apprenticeship, employee or civil servant) vs. not formally employed (all others)) and work stress (continuous variable) as potential confounders. Age and country of origin were not selected as confounders due to the homogeneity of our study population with respect to these variables. The rationale for including work stress as measured by the TICS score was to adjust for it as a proxy for other psychosocial exposures at work. As this approach may be regarded as a form of over-adjustment for a factor lying on the causal pathway between exposure and outcome, we performed an additional regression model without adjustment for work stress. All models calculated the odds ratio (OR) with 95% confidence interval (CI) as effect estimate with the lowest exposure group (0 types of experienced discrimination) as reference category.

Besides, we performed several sensitivity analyses. First, we repeated logistic regression with a different categorization of the predictor, i.e., four instead of three categories (0 types of experienced discrimination, 1 type, 2 types, 3 or more types). Secondly, we performed two subgroup analyses: one including only women (the number of male participants indicating multiple types of discrimination was too small to perform a subgroup analysis for men) and another one including only participants in formal employment. Additionally, we ran linear regression models with number of types of discrimination as (continuous) predictor and the PHQ-2 score (continuous) as outcome calculating the regression coefficient (B) and the corresponding 95% confidence interval (CI) as well as the standardized regression coefficient (β) as effect estimates (using the identical set of confounders as in the logistic regression models). Furthermore, we repeated all models after removal of one outlier that indicated having experienced all seven types of discrimination. To account for sparse data with respect to the exposure and outcome variables, we additionally performed a non-parametric bootstrapping procedure to estimate the standard errors and 95% confidence intervals for the logistic regression coefficients and odds ratios provided by the models with and without adjustment for work stress. We generated 1000 bootstrap replications by resampling the original dataset refitting the respective model for each bootstrap. Confidence intervals were obtained using the bias-corrected and accelerated (BCa) method.

For most variables, there were hardly any missing data (percentage of missing observations below 1%). Only for work stress, exposure to discrimination and depressive symptoms, the amount of missing observations was slightly higher (around 3%). Hence, we assumed that bias due to missing data is very unlikely and the benefits of performing complete-case analyses to outweigh the potential risks of multiple imputation such as specification error, particularly with respect to imputation of the outcome. All analyses were thus conducted as complete-case analysis using SPSS version 29.0 (IBM, Armonk, NY, USA).

## Results

### Characteristics of the study population

Participants’ mean age was 31.3 years (SD: 0.7), more than half of them (56.8%) were women and almost all of them (97.5%) were born in Germany. Furthermore, more than half of the participants had a university degree and more than 80% were currently employed. The most reported type of discrimination at work during the last twelve months was discrimination because of sex (6.1%). Among women, the prevalence of sex-related discrimination was 9.2%. Concerning work stress, participants on average reported low to moderate levels of stress (mean: 37.3 on a scale ranging from 0 to 88 points). While 88.7% of the individuals indicated no exposure to any type of discrimination, 26 participants (2.5%) reported that they had experienced two or more types of discrimination in the last year. Among the individuals reporting multiple types of discrimination, about two-thirds (65.4%) were women. With respect to the outcome, participants had a PHQ-2 mean score of 1.23 points (SD: 1.22) with 104 individuals (9.8%) being screened positive for depression (Table 1).

**Table 1:** Characteristics of the study participants (*N* = 1066)

Variable	Missing (*n*)	Mean (SD)	Minimum - Maximum
**Age (years)**	0	31.3 (0.7)	29–34
**Work stress** ^**a**^	21	37.3 (12.4)	5–81
**Depressive symptoms score (PHQ-2)** ^**b**^	20	1.23 (1.22)^c^	0–6
**Variable**	**Missing (n)**	**n**	**%**
**Sex**	0		
Female		605	56.8
Male		461	43.2
**Study center**	0		
Dresden		615	57.7
Munich		451	42.3
**Country of birth**	7		
Germany		1033	97.5
Other		26	2.4
**Educational attainment**	8		
No qualification		8	0.8
Vocational training (vocational school)		293	27.5
Vocational training (technical college)		120	11.3
University of applied sciences degree		78	7.3
University degree (Bachelor’s)		91	8.5
University degree (Master’s)		448	42.0
Other degree		9	0.8
Student		11	1.0
**Current form of occupation**	9		
Apprenticeship		14	1.3
Student		24	2.3
Employee		892	83.7
Civil servant		41	3.8
Self-employed		55	5.2
Job seeker		3	0.3
Not working for health reasons		3	0.3
Maternity protection / parental leave		25	2.3
**Discrimination**	32		
Because of Age		28	2.6
Because of Origin, Ethnic Background or Color		7	0.7
Because of Nationality		8	0.8
Because of Sex		65	6.1
Because of Religion		5	0.5
Because of Disability		10	0.9
Because of Sexual Orientation		2	0.2
None		946	88.7
**Number of types of experienced discrimination**	32		
0		946	88.7
1		62	5.8
2		19	1.8
3		6	0.6
7		1	0.1

^a^ based on 22 items (three scales) from the TICS (range: 0–88); ^b^ range: 0–6; ^c^ *n* = 104 individuals (9.8%) with score ≥ 3; PHQ-2, Patient Health Questionnaire-2; SD, standard deviation

The prevalence of depressive symptoms among the non-exposed participants was 8.2%, while it increased with the number of types of experienced discrimination, even though this observation is based on a very small absolute number of individuals (Table 2).

**Table 2:** Prevalence of depressive symptoms by number of types of experienced discrimination (*N* = 1026)

Number of types of experienced discrimination	Depressive symptoms^a^	
	No	Yes
0	861 (91.8%)	77 (8.2%)
1	49 (79.0%)	13 (21.0%)
2	13 (68.4%)	6 (31.6%)
3	2 (33.3%)	4 (66.7%)
7	1 (100%)	0 (0%)

^a^ based on PHQ-2 score using a cut-off of ≥ 3 points; missing observations: n=40; percentages are row percentages

### Experience of discrimination in association with depressive symptoms

Without adjustment for work stress, individuals reporting two or more types of discrimination at work had more than six times the odds of depressive symptoms compared to the non-exposed reference group (OR: 6.31, 95% CI: 2.67–14.89). Exposure to one type of discrimination was also associated with a positive depression screening (OR: 2.74, 95% CI: 1.38–5.46) (Table 3).

**Table 3:** Results from the logistic regression for the association between number of types of experienced discrimination and depressive symptoms before adjustment for work stress (*N* = 1020)

	OR	95% CI
Number of types of experienced discrimination		
0	Ref.	
1	2.74	1.38–5.46
2 or more	6.31	2.67–14.89
**Sex**		
Male	Ref.	
Female	1.00	0.65–1.55
**Study center**		
Munich	Ref.	
Dresden	0.80	0.52–1.23
**Educational attainment**		
University degree	Ref.	
No university degree	1.63	1.06–2.50
**Current form of occupation**		
Not formally employed	Ref.	
Formally employed	0.87	0.45–1.70

After adjustment for work stress, participants indicating two or more types of discrimination at work had almost four times the odds of depressive symptoms than individuals who had experienced no discrimination (OR: 3.92, 95% CI: 1.40 to 11.01; Table 4). The evidence for an association of one type of discrimination with depressive symptoms attenuated when adjusting for work stress (OR: 1.82, 95% CI: 0.86–3.82). Besides, an increase in the work stress score by one point was associated with an increase in the odds of depressive symptoms (OR: 1.12, 95% CI: 1.09–1.14) while participants without university degree were more likely to indicate depressive symptoms than participants with a university degree (OR: 1.81; 95% CI: 1.11–2.91). These effect estimates for the covariates should, however, not be over-interpreted as to avoid ‘Table 2 fallacy’ [52].

**Table 4:** Results from the logistic regression for the association between number of types of experienced discrimination and depressive symptoms after adjustment for work stress(*N* = 1018)

	OR	95% CI
Number of types of experienced discrimination		
0	Ref.	
1	1.82	0.86–3.82
2 or more	3.92	1.40–11.01
**Work stress**		
Total score (continuous)	1.12	1.09–1.14
**Sex**		
Male	Ref.	
Female	0.81	0.50–1.32
**Study center**		
Munich	Ref.	
Dresden	0.94	0.58–1.51
**Educational attainment**		
University degree	Ref.	
No university degree	1.81	1.12–2.91
**Current form of occupation**		
Not formally employed	Ref.	
Formally employed	1.46	0.68–3.13

Ref., reference category; OR, odds ratio; CI, confidence interval

In the subgroup analysis including only women, the OR for the association between two or more types of discrimination and depressive symptoms was 6.66 (95% CI: 1.89 to 23.45; Table 5). In the subgroup analysis including only participants in a formal employment relationship, exposure to two or more types of discrimination was also associated with the odds of depressive symptoms (OR: 3.77, 95% CI: 1.23 to 11.55; Supplement 1). When classifying the exposure variable into four categories (no discrimination compared to 1, 2 or ≥ 3 types of discrimination), the OR for the association between 2 types of experienced discrimination and depressive symptoms was 2.57 (95% CI: 0.71 to 9.30) while for 3 or more types of discrimination the effect estimate after adjustment for work stress was OR: 10.28 (95% CI: 1.62 to 65.04; Supplement 2). In the linear regression models, after adjustment for all potential confounders, an increase in the number of types of experienced discrimination was associated with an increase in the depressive symptoms score by a quarter point (B (95% CI): 0.256 (0.118 to 0.394); β = 0.098). Removing the outlier reporting all seven types of discrimination did not lead to a noteworthy change in the effect estimates in any of the models (data not shown). The OR for exposure to discrimination derived from the bootstrapping approach were identical with those provided by the ordinary models (exposure to 2 or more types of discrimination before adjustment for work stress: OR: 6.31, 95% BCa CI: 2.16–14.71; exposure to 2 or more types of discrimination after adjustment for work stress: OR: 3.92, 95% BCa CI: 1.38–10.42).

**Table 5:** Results from the logistic regression for the association between number of types of experienced discrimination and depressive symptoms including only female participants (*N* = 575)

	OR	95% CI
Number of types of experienced discrimination		
0	Ref.	
1	2.69	1.21–5.99
2 or more	6.66	1.89–23.45
**Work stress**		
Total score (continuous)	1.11	1.08–1.14
**Study center**		
Munich	Ref.	
Dresden	0.90	0.48–1.68
**Educational attainment**		
University degree	Ref.	
No university degree	2.44	1.28–4.65
**Current form of occupation**		
Not formally employed	Ref.	
Formally employed	1.39	0.55–3.49

Ref., reference category; OR, odds ratio; CI, confidence interval

## Discussion

The present analysis aimed to estimate the implications of multidimensional discrimination at work for the mental health of the affected individuals. To achieve this aim, we analyzed cross-sectional data from 1066 young adults living in Germany. Participants who reported experiences of multiple types of discrimination at work had elevated odds of depressive symptoms compared to their peers without exposure to discrimination. This result supports the hypothesis of an association between multidimensional discrimination at the workplace and reduced mental health and well-being even though our effect estimates suffer from low statistical precision and thus a certain degree of uncertainty.

Exposure to only one type of discrimination was associated with depressive symptoms solely in the subgroup analysis including women only. In all models, the effect estimates for multiple types of discrimination were larger than those for one type of discrimination. On the one hand, this observation might be interpreted as supporting the hypothesis that multiply discriminated groups are especially vulnerable [31]. On the other hand, as mentioned above, the effect sizes we observed should be interpreted with great caution since due to the limited absolute number of individuals indicating exposure to discrimination our effect estimates come with rather little statistical precision (as indicated by the wide confidence intervals).

### Alignment with the existing literature

The observed prevalence of depressive symptoms in our study population of 9.8% is slightly below the estimates for the entire German population in the time period 2017–2018 (around 12% in the age group 30–39 years [53]). The same holds true for the prevalence of discrimination as 8% of our participants reported at least one experience of workplace discrimination during the last year prior to the survey whereas the EWCS estimated the prevalence of workplace discrimination in Germany at 10% [20].

The observation of an association between exposure to workplace discrimination and depressive symptoms is in line with the results of other studies in the field. For example, a recent study in the US identified poor mental health levels among individuals with multiple marginalized statuses [54] while in another investigation individuals who experienced discrimination across multiple intersecting identities reported elevated rates of psychological distress [55]. In Germany, two other studies reported evidence for associations between discrimination and intersecting dimensions of social inequality with mental ill-health although both of them not explicitly referred to discrimination at work so that the comparability to our study is limited [56, 57]. However, a number of studies looking at workplace discrimination as risk factor for poor mental health yielded results comparable to ours, even though they mostly did not specifically account for multiple discrimination [16, 26, 58, 59]. Furthermore, as also outlined in the introduction, a link between exposure to workplace discrimination and impaired mental health appears biologically plausible.

### Limitations

Readers should keep in mind several caveats when interpreting our results. With a mean age of 31 years, 97% being born in Germany and more than half of them holding a university degree (compared to 37% in the German general population), the representativeness of our study population for the entire German population is very limited. This homogeneity of our study population is also the most likely explanation why our dataset contained hardly any reports of discrimination on the grounds of age, ethnicity, or religion. The prevalence of discrimination at work reported by our study participants therefore should not be mistaken as representative for the entire workforce in Germany.

In this context, it also needs to be highlighted that about half of the participants of the previous follow-up (SOLAR 2) did not take part in SOLAR 3 and there is indication from an initial data analysis that SOLAR 3 is to some extent biased towards a more female and highly educated study population (Forster et al., 2021). Unfortunately, data on participants’ mental health and exposure to discrimination was not collected before SOLAR 3. Consequently, we cannot rule out the possibility that particularly individuals with impaired mental health may have more likely to drop out of the study because of their symptoms than their healthy counterparts. In this case, we would have underestimated the true prevalence of depressive symptoms in our sample. Furthermore, it needs to be kept in mind that the present analysis included only individuals who had performed some form of occupation during the last twelve months, excluding individuals who were job-seeking or too sick to work. Therefore, the prevalence of symptoms of depression in the present study cannot be generalized the entire German population.

Discrimination because of gender was by far the most frequently reported type of discrimination among the seven types that we asked for in the questionnaire. Therefore, our results are mostly driven by this type of discrimination. Speaking of gender issues, it is a shortcoming of our study that this characteristic was only measured as a binary variable which admittedly may be seen as a form of discrimination of gender diverse participants. Hence, we do not know the proportion of participants identifying themselves as non-binary as well as the prevalence of workplace discrimination in this group (as German legislation approved the third option ‘diverse’ only shortly after our survey was performed, we reckon that non-binary individuals in our study population will have indicated the sex category (male/female) assigned at birth).

Furthermore, we also had no information on other characteristics shaping an individual’s social identity such as sexual orientation. In combination with the negligible number of individuals born outside Germany and the restriction to young adults, we could not analyze potential interacitons between specific types of discrimination.

We also have no information on the form by which participants had felt discriminated at work, i.e., whether it was via interpersonal relationships, hiring, promotion or salary practices, work assignments, access to training opportunities, etc [11]. Furthermore, while we took into account chronic work stress by means of three scales of the established TICS [50], we had no further details on participants’ psychosocial work environment such as specific work demands (e.g., emotional demands) or resources. Concerning the latter, there is some indication that support at organizational, managerial and interpersonal level (e.g., peer support) may help minoritized workers to avoid discrimination and to protect their mental well-being [60, 61]. We consider it as a worthwhile line of research for upcoming studies to create additional knowledge concerning resources that help to mitigate detrimental effects of workplace discrimination (even though of course the primary aim should be to abolish discrimination altogether).

The role of work stress in the association between workplace discrimination and depressive symptoms cannot be determined from these cross-sectional data. We therefore present estimates with and without additional adjustment for work stress without treating either specification as a definitive causal estimate. The attenuation after adjustment does not, by itself, establish mediation through work stress. Besides, we had no data on participants’ exposure to discrimination outside work so that we cannot preclude unknown confounding by this variable. As all our measures were based on participants’ self-reports, we cannot preclude that common method bias [62] and misclassification of the exposure and/or outcome may have impacted our results. Common method bias could have occurred, for instance, if a certain number of participants had the tendency to ‘over-state’ their responses both regarding exposure to discrimination and symptoms of depression. This would have biased our results towards an overestimation of the true effect of workplace discrimination on depression. The same applies to a scenario where participants who were misclassified as depressed also ‘over-stated’ their experiences of discrimination at work or vice versa (i.e., differential misclassification). Non-differential misclassification of exposure and/or outcome meanwhile could have potentially biased our results to any direction [63]. Finally, the cross-sectional nature of our analysis does not allow us to make causal conclusions regarding the effect of multi-discrimination at work on depressive symptoms, and we cannot fully preclude reverse causality. Concerning the latter, it appears not unplausible that some participants experiencing symptoms of depression could have been more likely to perceive or report social interactions as being discriminatory since negative affectivity is a major vulnerability factor for depression. Under this scenario, which could also be interpreted as a form of recall bias, our effect estimates would have overestimated the true association between exposure and outcome. This shortcoming maybe be overcome at least partly by future longitudinal studies.

As already mentioned, our findings suffer from limited statistical precision. The sample size, particularly the small number of exposed men, did not allow a proper assessment of between-group differences. Therefore, the higher point estimate among women compared to the entire study population should not be misinterpreted as conclusive evidence for a stronger association in women. Elucidating potential differences by gender in the association between workplace discrimination and mental ill-health may, however, be an interesting objective for future research.

There were very few missing observations in our dataset. We therefore complete-case analyses as very unlikely to introduce potential bias into our results. Nonetheless, due to the sensitive nature of being a victim of discrimination, among those participants not answering the respective item, there may have been some individuals who actually had experienced discrimination but were reluctant to report it. Under this scenario, our estimates could be biased towards an underestimation of discrimination. At the same time, given that information on exposure to discrimination was missing only for 32 out of 1066, we reckon that this form of bias, if present at all, could have only had a very small impact on our estimates.

Lastly, we acknowledge that our approach of summing up the number of types of discrimination ignores the possibility that there may be qualitative differences in the potential impact of different grounds of discrimination. Unfortunately, our sample size (particularly the low absolute number of individuals reporting discrimination) did not allow to disentangle potential specific effects of different types of discrimination or interaction between them.

### Practical implications

The available evidence as a whole clearly points towards the important role that anti-discrimination initiatives can play for preventing individual suffering and mental ill-health [26] including Diversity, Equity and Inclusion (DEI) measures at the workplace [24, 64, 65]. For instance, a recent study identified inclusive and non-discriminatory policies as one of the key components to promote well-being and positive work outcomes among transgender and gender diverse employees [60]. On the long run, it may be worthwhile to integrate programs fostering awareness about DEI already in professional training as it has been proposed, for instance, in medical education [66, 67].

We are aware that in times of a global rise of the far right whose protagonists actively promote and fuel stereotypes, prejudice and discrimination in their most blatant forms like racism, xenophobia, misogyny or homophobia as part of their political agenda, there is a considerable backlash against DEI in many societies [64, 68]. Looking at the scientific evidence, it becomes clear that divisive and discriminatory policies come at a considerable price in form of severe consequences for individual and population health [3, 69–71] with anti-DEI measures leading to an increase in social and health inequities [72, 73]. Therefore, we advocate for strengthening DEI and anti-discriminatory initiatives instead of weakening them as they are valuable means of promoting public health and reducing health inequalities [68, 69].

## Conclusion

In the present study conducted among young adults living in Germany, participants who reported experiences of multiple types of discrimination at work had elevated odds of a positive depressive symptoms screening compared to their counterparts not reporting exposure to workplace discrimination. These results support the hypothesis of an association between exposure to multiple discrimination at work and poor mental health, even though our effect estimates are limited in their statistical precision, thus suffering from a noteworthy degree of uncertainty. It also needs to be kept in mind that the design of our analysis does not allow to make causal statements. In light of the entire scientific body of investigations into workplace discrimination and their health implications, we argue that measures to reduce or abolish discrimination may be seen as potentially valuable means of protecting mental health and well-being.

## Supplementary Information

Below is the link to the electronic supplementary material.

Supplementary Material 1

## Data Availability

The datasets presented in this article are not readily available because of data protection reasons. Requests to access the datasets should be directed to the corresponding author.
