# Supplementary material for: Multiple discrimination at work and depressive symptoms in young adults
Source: BMC Psychol. 2026 Oct 11;14:1463. doi: 10.1186/s40359-026-05765-y (PMC13654232; doi:10.1186/s40359-026-05765-y)
Supplement: Supplementary file 1 [file 40359_2026_5765_MOESM1_ESM.docx]

**Supplementary Materials**

Supplement 1: Sub-group analysis including only participants in formal employment

Table S1: Results from the logistic regression for the association between number of types of experienced discrimination and depression including only participants in formal employment (N=947)

|  | **OR** | **95% CI** |
| --- | --- | --- |
| **Number of types of experienced discrimination** | | |
| 0 | Ref. | |
| 1 | 2.09 | 0.97 -4.49 |
| 2 or more | 3.77 | 1.23 – 11.55 |
| **Work stress** | | |
| TICS total score (continuous) | 1.12 | 1.09 -1.14 |
| **Sex** |  |  |
| Male | Ref. | |
| Female | 0.78 | 0.46-1.30 |
| **Study center** | | |
| Munich | Ref. | |
| Dresden | 1.00 | 0.61-1.66 |
| **Educational attainment** | | |
| University degree | Ref. | |
| No university degree | 1.75 | 1.06-2.90 |

Ref., reference category; OR, odds ratio; CI, confidence interval

Supplement 2: Logistic regression with four exposure categories

Table S2: Results from the logistic regression for the association between number of types of experienced discrimination (four categories) and depression (N=1018)

|  | **OR** | **95% CI** |
| --- | --- | --- |
| **Number of types of experienced discrimination** | | |
| 0 | Ref. | |
| 1 | 1.81 | 0.86-3.82 |
| 2 | 2.57 | 0.71-9.30 |
| 3 or more | 10.28 | 1.62-65.04 |
| **Work stress** | | |
| Total score (continuous) | 1.12 | 1.09 -1.14 |
| **Sex** | | |
| Male | Ref. | |
| Female | 0.81 | 0.50-1.32 |
| **Study center** |  |  |
| Munich | Ref. | |
| Dresden | 0.95 | 0.59-1.53 |
| **Educational attainment** | | |
| University degree | Ref. | |
| No university degree | 1.76 | 1.09-2.84 |
| **Current form of occupation** | | |
| Not formally employed | Ref. | |
| Formally employed | 1.50 | 0.69-3.24 |

Ref., reference category; OR, odds ratio; CI, confidence interval;
